# Treatment of severe acute ulcerative colitis in SARS-CoV-2 infected patients: report of three cases and discussion of treatment options

**DOI:** 10.1177/17562848211012595

**Published:** 2021-04-29

**Authors:** Arno R. Bourgonje, Reinier C. A. van Linschoten, Rachel L. West, Maarten A. van Dijk, Coretta C. van Leer-Buter, Gursah Kats-Ugurlu, Marieke J. Pierik, Eleonora A. M. Festen, Rinse K. Weersma, Gerard Dijkstra

**Affiliations:** Department of Gastroenterology and Hepatology, University of Groningen, University Medical Center Groningen, Hanzeplein 1, Groningen, 9700 RB, the Netherlands; Department of Gastroenterology and Hepatology, Franciscus Gasthuis & Vlietland, Rotterdam, the Netherlands; Department of Gastroenterology and Hepatology, Franciscus Gasthuis & Vlietland, Rotterdam, the Netherlands; Department of Gastroenterology and Hepatology, Elkerliek Hospital, Helmond, the Netherlands; Department of Medical Microbiology, University of Groningen, University Medical Center Groningen, Groningen, the Netherlands; Department of Pathology and Medical Biology, University of Groningen, University Medical Center Groningen, Groningen, the Netherlands; Department of Gastroenterology and Hepatology, University of Maastricht, University Medical Center Maastricht, Maastricht, the Netherlands; Department of Gastroenterology and Hepatology, University of Groningen, University Medical Center Groningen, Groningen, the Netherlands; Department of Gastroenterology and Hepatology, University of Groningen, University Medical Center Groningen, Groningen, the Netherlands; Department of Gastroenterology and Hepatology, University of Groningen, University Medical Center Groningen, Groningen, the Netherlands

**Keywords:** ulcerative colitis, IBD, COVID-19, SARS-CoV-2, case series

## Abstract

In the wake of the coronavirus disease 2019 (COVID-19) pandemic, it is unclear how asymptomatic severe acute respiratory syndrome coronavirus-2 (SARS-CoV-2)-infected patients who present with acute severe ulcerative colitis (UC) can be treated effectively and safely. Standard treatment regimens consist of steroids, immunomodulatory drugs, and biological therapies, but therapeutic decision-making becomes challenging as there are uncertainties about how to deal with these drugs in patients with COVID-19 and active UC. Importantly, guidelines for this particular group of patients with UC are still lacking. To inform therapeutic decision-making, we describe three consecutive cases of patients with active UC and COVID-19 and discuss their treatments based on theoretical knowledge, currently available evidence and clinical observations. Three patients were identified through our national inflammatory bowel disease network [Initiative on Crohn’s and Colitis (ICC)] for whom diagnosis of SARS-CoV-2-infection was established by reverse transcription–polymerase chain reaction (RT-PCR) testing in nasopharynx, stools, and/or biopsies. Acute severe UC was diagnosed by clinical parameters, endoscopy, and histopathology. Clinical guidelines for SARS-CoV-2-negative patients advocate the use of steroids, calcineurin inhibitors, or tumor necrosis factor alpha (TNF-α)-antagonists as induction therapy, and experiences from the current three cases show that steroids and TNF-α-antagonists could also be used in patients with COVID-19. This could potentially be followed by TNF-α-antagonists, vedolizumab, or ustekinumab as maintenance therapy in these patients. Future research is warranted to investigate if, and which, immunomodulatory drugs should be used for COVID-19 patients that present with active UC. To answer this question, it is of utmost importance that future cases of patients with UC and COVID-19 are documented carefully in international registries, such as the SECURE-IBD registry.

## Introduction

Patients with inflammatory bowel diseases (IBD), encompassing Crohn’s disease (CD) and ulcerative colitis (UC), are at increased risk of serious viral infections, mainly because of uncontrolled disease activity and the use of immunosuppressive drugs.^1^ Patients with IBD often receive treatment with immunomodulating drugs, including steroids, thiopurines and methotrexate, JAK-STAT inhibitors, and biologicals targeting tumor necrosis factor alpha (TNF-α), interleukin (IL)-12/IL-23 or α4β7-integrin, which may increase the risk of viral infection. Both the higher risk of contracting viral infections and the use of immunomodulating drugs could theoretically render IBD patients particularly susceptible to coronavirus disease 2019 (COVID-19). A study from Italy reported that active disease, older age, and comorbidities were risk factors associated with a worse COVID-19 outcome in patients with IBD, whereas concomitant use of biologicals and other immunomodulating drugs were not.^2^ A recent report from the international SECURE-IBD registry showed that thiopurine monotherapy and combination therapy with thiopurines and TNF-α-antagonists were associated with an increased risk of severe COVID-19 compared with TNF-α-antagonist monotherapy.^3^ Similarly, aminosalicylates showed a slightly increased risk for severe COVID-19. These data have led to an ongoing discussion on starting and continuation of immunomodulating treatment in patients with IBD.^4^ Although SARS-CoV-2 infects the gastrointestinal mucosa, there is no conclusive evidence for an increased risk of aggravated outcome in patients with IBD affected by COVID-19.^4^ Recently, we showed that patients using TNF-α-antagonists and those with active mucosal inflammation show increased intestinal expression of the SARS-CoV-2 host protease transmembrane protease, serine 2 (TMPRSS2), indicating a potential higher susceptibility of SARS-CoV-2 infection in these patients.^5^ Nonetheless, due to the lack of original research data, there is still much uncertainty about the implications of COVID-19 for patients with UC, especially for those with active disease.^6^ Most importantly, guidelines covering this subgroup of patients are inconclusive at this moment, necessitating consensus-based recommendations in the absence of more evidence-based data.^7–9^

In this report, we describe three consecutive patients with UC in the Netherlands who contracted COVID-19 and presented clinically with either a first presentation or flare of acute severe UC. Furthermore, we highlight the effectiveness and safety of their treatment regimens. Based on our clinical experience, we aim to propose suitable management strategies for patients with active ulcerative colitis who develop COVID-19 without pulmonary symptoms.

## Case presentations

**Figure 1.**
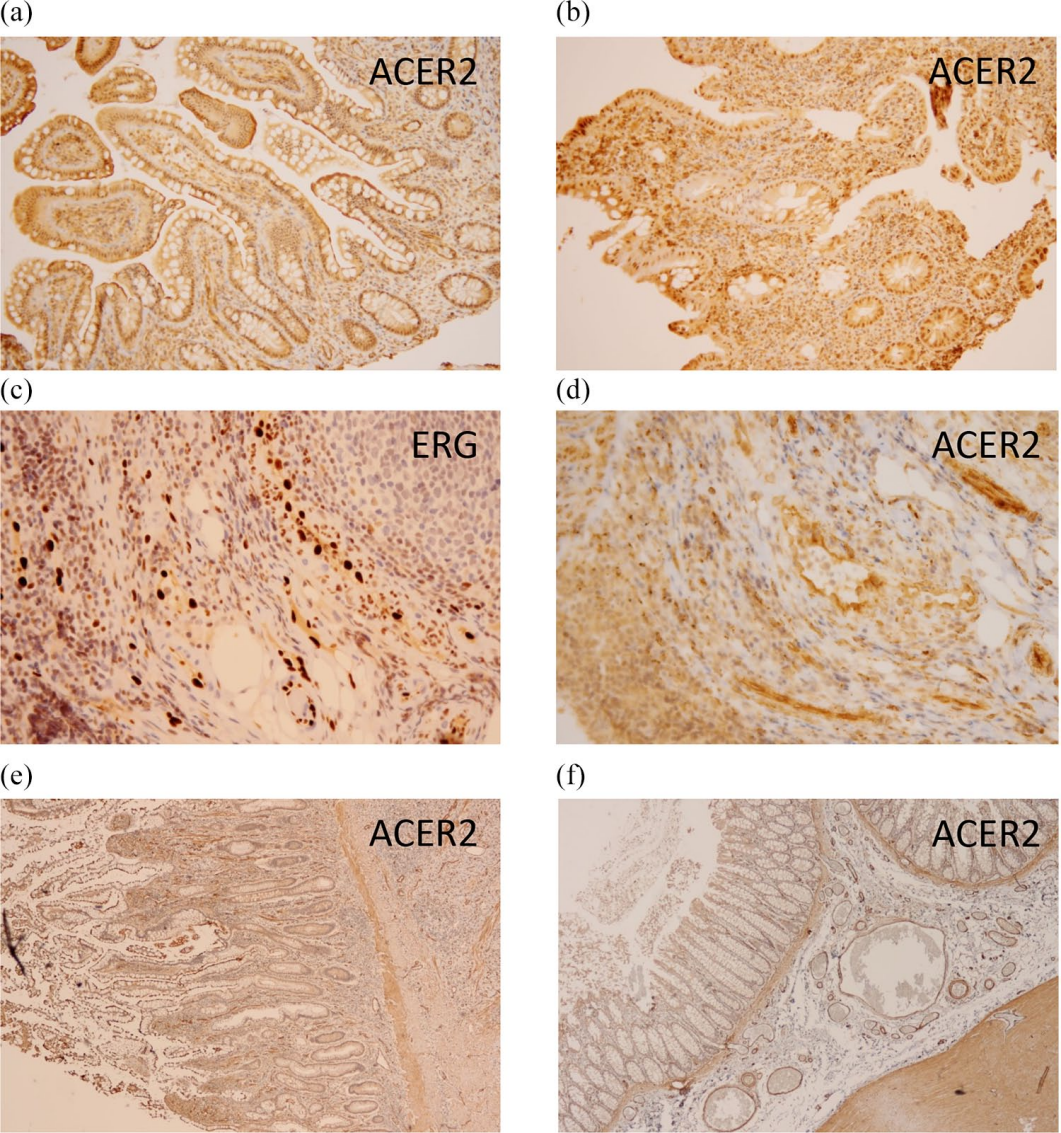
Histopathology of terminal ileum and transverse colon biopsies with staining for ACER2 and the vascular endothelial marker ERG. Terminal ileum biopsies showed mucosae without distortion of villi- or crypt architecture. There was slight expansion of the inflammatory infiltrate, which consisted of plasma cells, lymphocytes, eosinophilic granulocytes, and a few neutrophils. In addition, there were few lymphoid aggregates and a lymphoid follicle. There were no granulomas or epithelial dysplasia. The additional ACE2 staining showed enhanced staining at the brush border and in the vascular endothelium (a, e). Transverse colon biopsies showed slight distortion of the villous architecture of the surface epithelium and slight edema with focal epithelial detachment. There was expansion of the inflammatory infiltrate, which consisted mainly of plasma cells, and neutrophilic-, and eosinophilic granulocytes. There were signs of cryptitis and crypt abscesses. Some crypts showed no architectural changes whereas others showed partial or total damage. Interestingly, the small capillaries showed endotheliitis resulting from extravasation of neutrophilic granulocytes. These activated prominent endothelial cells showed no fibrin deposition or damage. The additional ACE2 staining showed no enhanced staining at the brush border and the staining in the vascular endothelium was blurry (b, d, f). Endothelial cells are highlighted in the ERG staining (c). ACE, angiotensin-converting enzyme 2; ACER2, angiotensin-converting enzyme receptor 2; ERG, ETS-related gene.

Patient A (SARS-CoV-2 infection and *de novo* pancolitis) was a 25-year-old male who experienced abdominal cramps and a feeling of raised body temperature (unmeasured) for a period of several days. Suddenly, he developed a fever (>38.3°C) and complained of headaches with a concurrent increase in abdominal cramps and diarrhea. After 3–4 days, he was admitted to the emergency department because of diarrhea with rectal bleeding. Upon admission, the patient was normothermic (36.8°C), but had mild tachycardia and a stool frequency of seven times a day. Laboratory examination showed a C-reactive protein (CRP) level of 6 mg/l (N: <5 mg/l), hemoglobin level of 8.6 mmol/l (N: >8.0 mmol/l), and lymphocyte count of 1.4 × 10^9^/l (N: 1.0-4.0 × 10^9^/l). Initial diagnostic work-up with stool cultures and throat swabs was commenced while he received prophylactic low-molecular weight heparins (LMWH) and empirical antibiotic treatment with ciprofloxacin under suspicion of a gastrointestinal infection. He was re-evaluated 2 days later and had persistent bloody diarrhea, but without respiratory symptoms or fever (36.1°C). New laboratory examination showed an elevated CRP (18 mg/l) and a lymphocyte count of 1.6 × 10^9^/l. A chest X-ray was performed which showed no abnormalities. Prior stool cultures were negative, while both stool, nose, and throat samples were positive for SARS-CoV-2 RNA using real-time reverse transcription–polymerase chain reaction (rRT-PCR) testing. The patient was diagnosed with infectious diarrhea of unknown origin (potentially SARS-CoV-2 related) and was discharged from the hospital since his frequency of diarrhea decreased. However, he was soon readmitted because of recurrent bloody diarrhea and abdominal pain and cramps. Similar to the previous evaluation, he had no fever and a chest X-ray was normal. Laboratory examination showed leukocytosis (15.4 × 10^9^/l, N: 4.0–10 × 10^9^/l), thrombocytosis (485 × 10^9^/l, N: 100–400 × 10^9^/l), increased fibrinogen (5.8 g/l, N: 2.0–4.0 g/l), elevated CRP (117 mg/l), and negative serology for Epstein–Barr virus (EBV) and cytomegalovirus (CMV). Second testing for SARS-CoV-2 was positive in nose/throat swabs and viral RNA was found in stools. Endoscopic examination was performed and showed a pancolitis corresponding to a Mayo 3 severity score. Biopsies were taken from the terminal ileum and transverse colon (Figure 1). Pathologic findings from colonic biopsies (consisting of chronically active inflammation with epithelial damage, crypt abscesses, micro-abscesses and focal loss of crypt architecture) were compatible with the diagnosis of UC.

The patient was treated with prednisolone 40 mg intravenously daily, a considerable lower dosage than usual, as there was still uncertainty surrounding high-dose corticosteroid treatment. Additionally he was treated with mesalamine 4.8 g daily with the intention of quickly enabling tapering of corticosteroid treatment. Hydroxychloroquine was also administered according to the protocol at that time, when it was considered potentially effective in COVID-19, with a 600 mg loading dose, 300 mg after 12 h, and 300 mg twice daily for the next 5 days, after which all of his clinical symptoms eventually resolved. However, 23 days after the start of mesalamine treatment, he was readmitted because of chest pain. Throat swabs were still positive for SARS-CoV-2, while a diagnosis of perimyocarditis was established, most likely mesalamine-induced, as it recovered rapidly after discontinuation of mesalamine. A few days later, when the patient was already discharged from the hospital because of his rapid recovery, the patient contacted his treating physician with symptoms of recurrent bloody diarrhea and abdominal discomfort. These symptoms were compatible with a flare of colitis at a time the patient tapered the corticosteroids to just prednisolone 25 mg daily according to the prescribed steroid regimen. Eventually, throat swab and intestinal biopsies were negative for SARS-CoV-2. Subsequently, vedolizumab 300 mg following a standard induction therapy regimen (0–2–6–14 weeks) was initiated and the patient achieved both clinical and endoscopic remission upon this treatment, and steroids could be tapered successfully and eventually stopped.

Patient B (SARS-CoV-2 infection and *de novo* pancolitis) was a 35-year-old male who presented to the emergency department with clinical symptoms of abdominal pain, diarrhea since 1 week and rectal blood loss since 2 weeks. Additionally, he had a fever (38.5°C), but no respiratory symptoms. Laboratory examination showed strongly elevated CRP levels (224 mg/l) and leukocytosis (15.7 × 10^9^/l). Stool cultures and testing for *Clostridium* *difficile* yielded negative results. Sigmoidoscopy revealed the presence of severe continuous colitis (Mayo 3) up to the transverse colon. After endoscopy, his fever aggravated (38.8°C) and he developed a sore throat. Treatment with prednisolone was withheld awaiting RT-PCR results for SARS-CoV-2. At that time, he tested positive for SARS-CoV-2 and treatment with both oral and rectal mesalamine was started instead of high-dose corticosteroids, as there was a lot of uncertainty regarding their use at the beginning of the pandemic. His clinical status deteriorated with signs of tachycardia (113/min), abdominal pain, bloody diarrhea, and increased CRP levels (316 mg/l), and an abdominal X-ray showed no toxic megacolon. Intravenous prednisolone (40 mg intravenously) was administered for 3 days, as well as prophylactic LMWH, but then his clinical status deteriorated further with tachycardia (120/min), increasing abdominal pain, and high stool frequency (12 times/day). Rescue therapy with the calcineurin inhibitor cyclosporine was considered, but a second abdominal X-ray revealed signs of a toxic megacolon with a distended colon of >7 cm. The patient immediately underwent subtotal colectomy with the construction of an ileostomy. Histopathology results after subtotal colectomy revealed signs of chronic inflammation with distortion of epithelial architecture and ulcerations, compatible with UC. Colonic perforation occurred during surgery, and the patient was transferred to the ICU because of a complicated postoperative course with persistent fever, tachycardia, and respiratory complaints. A chest X-ray showed pleural fluid collections and pulmonary infiltrates, suggestive of pneumonia as a result of his complicated surgery. Abdominal computed tomography (CT)-images revealed the presence of abdominal fluid. The patient was treated with antibiotics (cefuroxime) and gradually recovered in the following days.

### Histopathology

Biopsies of the colon showed hyperplastic changes of the epithelium. Crypts were ordered irregularly, but there was no clear distortion of the crypt architecture. Diffuse influx of neutrophilic granulocytes and crypt abscesses were seen. There was a diffuse increase of lymphoplasmacellular inflammatory infiltrate in the lamina propria with some eosinophilic granulocytes. Basal plasmacytosis was also observed.

Histopathology of the colon after resection showed extensive architectural changes, with an irregular surface and branching crypts. The entire colon showed signs of inflammation, while the resection plane of the ileum showed no such signs. There was evidence of basal plasmacytosis and infiltration of neutrophilic granulocytes around the epithelium. Ulcerations were seen, reaching to the muscularis propria. There were some focal abscesses and crypt abscesses. In conclusion, there were clear signs of chronic active inflammation with marked architectural distortion and ulcerations alongside the entire colon, which were considered compatible with the diagnosis of UC.

Patient C (SARS-CoV-2 infection and UC flare) was a 40-year-old female with an established diagnosis of distal UC existing for >15 years and was treated with local mesalamine maintenance therapy. She presented with complaints of abdominal cramps and increased stool frequency. Initial diagnostic evaluation by stool cultures and testing for *C. difficile* were negative. The patient returned 5 days later with bloody stools 10 times a day, tachycardia, and fever (38.0°C). Routine assessment for SARS-CoV-2 infection with rRT-PCR on a throat swab yielded a negative result. Laboratory examination showed an elevated CRP level of 92 mg/l and anemia (hemoglobin level 7.1 mmol/l). Abdominal X-ray examination showed no signs of toxic megacolon. The patient was hospitalized and underwent endoscopic investigation up to the splenic flexure showing severe continuous colitis (Mayo 3) with rectal sparing, possibly due to the use of mesalamine suppositories. The patient fulfilled the Truelove and Witt’s criteria for acute severe UC and treatment was initiated with prednisolone 40 mg intravenously and prophylactic LMWH. After 3 days of treatment, there was little clinical improvement with a bloody stool frequency of seven times a day and an elevated CRP level of 60 mg/l. Consequently, rescue therapy was initiated with an infliximab infusion (5 mg/kg) followed by a second infusion 7 days later. Despite treatment, she had a persistent fever (38.0 °C) and developed a dry cough. Chest CT showed no signs of viral pneumonia or pulmonary embolism. A rRT-PCR on a second throat swab was positive for SARS-CoV-2. At the time COVID-19 was diagnosed, stool frequency had decreased to one to two times a day and CRP level dropped to 27 mg/l. Upon clinical improvement, prednisone dosage was decreased to 30 mg orally. Clinical resolution followed 5 days later, with a resolved dry cough, normal body temperature, and a stool frequency one to two times a day without blood.

## Discussion and conclusions

In this report, we presented three consecutive cases of either *de novo* acute severe UC or flares of UC concurrent with a SARS-CoV-2 infection. Patient A responded well to treatment with steroids, and patient C responded well to the TNF-α-antagonist infliximab. However, for patient B, steroid treatment was delayed because of uncertainty surrounding steroids and SARS-CoV-2 infection, necessitating surgical intervention. All cases had a rRT-PCR confirmed SARS-CoV-2 infection, although none of the patients developed severe COVID-19 with respiratory involvement.

Patients with COVID-19 commonly experience gastrointestinal (GI) symptoms, including anorexia, diarrhea, vomiting, and abdominal pain.^10^ Patients experiencing GI symptoms have been observed to have a longer duration from disease onset to hospital admission and present with prolonged coagulation times and elevated liver function tests.^11^ In addition, the presence of viral RNA has repeatedly been confirmed in stools of patients with COVID-19, even after viral RNA could no longer be detected in the respiratory tract.^12,13^ It has been hypothesized that SARS-CoV-2 could invade the intestinal epithelium directly, suggesting a potential route of fecal–oral transmission.^10,11,14^ In a single-cell transcriptome study, high co-expression of angiotensin-converting enzyme 2 (ACE2) and TMPRSS2 was found to be present in absorptive enterocytes derived from both ileum and colon.^14^ Concomitant upregulation of ACE2 and TMPRSS2 in the inflamed intestinal mucosa could facilitate viral entry and provide an explanation to the findings of a study that demonstrated that active disease was associated with a worse outcome of COVID-19.^2,5^ In contrast, a recent report demonstrated that reduced ileal ACE2 expression was associated with inflammation and worse outcome of COVID-19 in patients with CD.^15^ These findings underline the paradoxical role of ACE2 as it may or may not implicate activation of downstream anti-inflammatory and anti-fibrotic effects, acting as protective mechanism in active IBD. However, further research into the role of ACE2 and TMPRSS2 in IBD and COVID-19 is warranted, for example through the comparison of viral loads in patients with quiescent versus active disease and the relationship with ACE2 expression and activity. Moreover, ACE2 has also been shown to be highly expressed in intestinal endothelium, and in vascular and intestinal smooth muscle cells.^16^ After initial viremia, SARS-CoV-2 may proceed to affect other organs after passage through the endothelium, including that of the GI tract, giving rise to endotheliitis as observed in Patient A.^17^ Patients with active UC may also be vulnerable to the reverse of this phenomenon, where SARS-CoV-2 may pass through the endothelium of the GI tract into the bloodstream, considering that inflamed intestinal mucosa is accompanied by endothelial injury and increased vascular permeability, as was evident in Patient A.

Patients A and B as described in this report raise the possibility that SARS-CoV-2 infection may precipitate *de novo* UC. For instance, one could hypothesize that SARS-CoV-2 may induce inflammation of the GI tract that results in an uncontrolled mucosal immune response, which cannot be sufficiently suppressed and culminates in persistent intestinal inflammation. However, the simultaneous occurrence of both COVID-19 and UC makes it hard to determine whether clinical, biochemical, endoscopic, and histological findings were related to UC, SARS-CoV-2 infection or a combination of both. Few case reports have described the co-occurrence of acute severe UC and COVID-19.^18–22^ Future studies are warranted to assess the potential association between COVID-19 as a causal trigger of UC.

Remarkably, Patient A developed perimyocarditis 23 days after starting mesalamine therapy, which quickly resolved after discontinuing this drug. Perimyocarditis is a rare complication of mesalamine therapy, which usually occurs within 28 days of commencing treatment. Although the underlying mechanism is unknown, it is hypothesized that a cell-mediated hypersensitivity reaction, rather than direct cardiotoxic injury, causes the perimyocarditis.^23,24^ However, the observed perimyocarditis might also have been caused by SARS-CoV-2, as it has frequently been reported as a potential consequence of SARS-CoV-2-associated myocardial injury.^25,26^ Cardiac pericytes express high levels of ACE2 and have been proposed as important SARS-CoV-2 target cells, resulting in the hypothesis that this may cause capillary endothelial cell dysfunction, which culminates in myocardial injury or perimyocarditis.^27^

To date, there are few evidence-based recommendations with regard to the use of immunosuppressive drugs in patients with IBD who are affected by COVID-19. Generally, it is advised that both adult and pediatric patients should not preemptively discontinue treatment as potential risks associated with pausing immunomodulation outweigh the risk of contracting COVID-19 or developing a severe outcome when infected with SARS-CoV-2.^28–30^ Similarly, patients with IBD who are treated with TNF-α-antagonists or other compounds targeting cytokine pathways, do not automatically seem to be conferring an increased risk of developing severe COVID-19.^31^ Although these medications may be considered immunosuppressive drugs, and therefore potentially harmful in the context of COVID-19, they specifically target individual pro-inflammatory cytokines or mediators and do not inhibit a broad range of immune system components. In addition, cytokine inhibitors are seriously considered as encouraging treatment options for COVID-19 as they are likely to attenuate the hyperinflammatory response associated with the disease.^17^ When evaluating the potential risks of biological therapies, it is important to examine which cytokine pathways are blocked as most of these substances are involved in the host inflammatory response and not mainly in viral clearance mechanisms.^4,31^

### Treatment recommendations for patients with acute severe UC and COVID-19

In the cases presented, several drugs could theoretically have been applied to influence the clinical course of patients with acute severe UC with COVID-19, including corticosteroids, calcineurin inhibitors (CNIs), TNF-α antagonists (e.g., infliximab), and vedolizumab. Here, we aim to highlight these medical treatments by integrating their known efficacy in UC with the currently available evidence for potential effectiveness in COVID-19 (Table 1).

**Table 1.** Potentially effective drugs in COVID-19 cases of patients with acute severe UC.

Drugs used in acute severe UC	Known efficacy in acute severe UC	Current evidence for potential effect in COVID-19
Corticosteroids	The mainstay of rescue therapy in patients with moderate-to-severe UC experiencing a disease flare	Dexamethasone (*n* = 2104) reduced 28-day all-cause mortality *versus* patients receiving standard of care (*n* = 4321), 21.6% *versus* 24.6%, age-adjusted rate ratio, 0.83[95%CI: 0.74–0.92], *p* < 0.001.^32^
		Methylprednisolone was associated with reduced risk of death [HR 0.38 (95%CI: 0.20–0.72)].^33^
Calcineurin inhibitors (e.g., cyclosporine, tacrolimus)	Effective in some cases of corticosteroid-refractory UC	CNIs have proven to be beneficial in case of other highly pathogenic coronaviruses, and might be of potential value in the treatment of COVID-19, though these observations are based primarily on experimental evidence.^34–37^
		Clinical course of COVID-19 in patients using CNIs seems to be relatively mild, with a low risk of superinfection.^38^
		Switching to cyclosporine-based immunosuppression may be an alternative therapeutic option in COVID-19 infection following solid organ transplantation.^39^
TNF-antagonists (e.g., infliximab, adalimumab, golimumab)	The first biological response modifiers to be used in the treatment of moderate to severely active UC, with very high response and remission rates.	Data from the SECURE-IBD registry showed that patients on TNF-α-antagonists therapy experienced lower rates of severe COVID-19 compared with non-users. However, in multivariable analysis, TNF-α antagonist therapy was not significantly associated with severe COVID-19.^3^
		A case report showed mild COVID-19 symptoms in an infliximab-treated UC patient, which suggested that ongoing anti-TNF therapy might protect against the viral hyperinflammatory response and avoid aggravated outcome.^40^
		Another case report described a patient with Crohn’s ileitis being treated with adalimumab and had a mild course of COVID-19 and rapid hospital discharge.^41^
Vedolizumab	Mostly used as second-line biological with proven effectiveness in the treatment of moderate to severely active UC	No evidence available.

CI, confidence interval; CNI, calcineurin inhibitor; COVID-19, coronavirus disease 2019; HR, hazard ratio; TNF-α, tumor necrosis factor alpha; UC, ulcerative colitis.

#### Corticosteroids

Corticosteroids systemically suppress the inflammatory response and may be beneficial in patients with both active IBD and COVID-19, as the latter may be accompanied by a systemic ‘cytokine storm’ aggravating the disease by inducing lung injury and widespread tissue damage. However, patients using corticosteroids are known to be notoriously prone to many opportunistic and respiratory infections, including influenza and coronavirus infections, such as SARS-CoV-1 and MERS-CoV infections.^42–44^ In addition, corticosteroid use is associated with an increased risk of hospitalization and mortality in patients with IBD, especially when higher dosages are administered.^45^ Several recommendations state that patients with IBD should avoid the use of corticosteroids or taper the use of corticosteroids as much as possible.^28,46^ Recently, preliminary results from the RECOVERY trial revealed that patients with severe COVID-19 benefited from receiving 6 mg dexamethasone once daily (*n* = 2104) as they had a significantly lower mortality rate compared with patients receiving standard of care (*n* = 4321).^32^ In this trial, dexamethasone was particularly beneficial for patients who were about to develop acute respiratory distress syndrome (ARDS). However, dexamethasone did not benefit patients with milder disease or those of older ages, and issues of optimal dosage, timing, or duration of therapy remained unaddressed.^47^ Likewise, it remains unclear whether patients with COVID-19 using corticosteroids for other medical conditions are at increased risk of adverse disease outcomes. Therefore, a potential, yet unknown, risk of developing an adverse outcome of COVID-19 should be balanced against the known efficacy of corticosteroids in treating disease exacerbations in patients with active UC. Based on the above, rapid tapering of corticosteroid treatment would be a preferred strategy as soon as disease activity decreases, considering the side-effects, increased risk of viral infections, and associations with higher hospitalization and mortality rates. If a patient with active UC and concomitant COVID-19 requires corticosteroid treatment, it is recommended to commence locally acting, high-dose corticosteroids (budesonide or beclomethasone) as these are characterized by fewer side effects.^46^

#### Calcineurin inhibitors

CNIs, cyclosporine or tacrolimus, are widely used immunosuppressants and are known to inhibit the production of IL-2 and the expression of its receptor (IL-2R), resulting in a decrease in T-cell activation. These agents are the cornerstone of treatment for patients who have undergone organ transplantation, but they are also effective in a fraction of patients with corticosteroid-refractory IBD. Interestingly, CNIs may inhibit viral replication. For instance, cyclosporine has been demonstrated to possess antiviral activity against several coronaviruses, including SARS-CoV-1.^33,34^ Genomic analysis of interactions between SARS-CoV-1 and human proteins previously identified cyclophilin family members and tacrolimus (FK506)-binding proteins as candidate interaction molecules for SARS-CoV-1-derived proteins.^35^ In support of this view, *in vitro* knockdown of FK506-binding proteins and FK506 treatment suppressed SARS-CoV-1 viral replication.^36^ Although these findings are all of experimental origin, they may provide arguments to either start CNI therapy in patients with active IBD or to continue preexistent maintenance treatment in patients with IBD who develop COVID-19. Induction therapy with CNIs may show clinical effectiveness in patients with steroid-refractory acute severe UC, followed by vedolizumab as maintenance treatment.^48^ CNIs might therefore be a potential alternative treatment to corticosteroids, if these would later appear to have more detrimental than beneficial effects in patients with active IBD and COVID-19.

#### TNF-α antagonists

TNF-α antagonists are known to convey an increased risk of serious respiratory viral infections, which further increases when combined with other immunosuppressive agents, especially corticosteroids.^49,50^ However, it is uncertain what the potential risks or benefits are of TNF-α antagonists in developing severe COVID-19 in patients with UC. Currently, there is no evidence for an increased risk of SARS-CoV-2 infection in patients with IBD.^28^ A report from the SECURE-IBD registry indicated that monotherapy with TNF-α antagonists does not appear to be a risk factor for severe COVID-19, and it may even have a protective effect against severe COVID-19.^51^ A recent study demonstrated increased serum TNF-α levels in patients with severe COVID-19, which may partially occur through increased ACE2 and/or TMPRSS2 expression in the inflamed intestinal epithelium.^3,4,52,53^ In contrast, however, TNF-α antagonist therapy has also been associated with restoration of intestinal ACE2 levels, particularly in TNF-α antagonist responders, suggesting a potential modulatory role of TNF-α antagonists in patients with IBD and concurrent COVID-19.^15^ In this way, TNF-blockade could foster ACE2 activity and thereby contribute to the resolution of inflammation associated with active IBD, as well as the exaggerated inflammatory response as is observed in some patients with COVID-19.

#### Vedolizumab

Long-term follow-up studies of patients with IBD receiving vedolizumab treatment have demonstrated that there is no increased risk of severe respiratory viral infections or severe (opportunistic) infections in general.^54–56^ In this respect, the use of vedolizumab in active IBD seems to be relatively safe. Previously, it has been demonstrated that COVID-19 is associated with endotheliitis in various organs, including the GI tract.^57^ It is suggested that direct infection of intestinal endothelium, resulting in endothelial dysfunction and perivascular inflammation, may culminate in intestinal microvascular pathology.^15^ Vedolizumab, which is a monoclonal antibody targeting α4β7-integrin, blocks leukocyte trafficking to the intestinal mucosa, and may limit transendothelial passage of immune cells in the context of endothelial dysfunction in patients with COVID-19. However, the administration of vedolizumab is usually performed in cases with more subacute manifestations of active UC or as a next treatment step after successful rescue therapy of acute severe UC.

### Concluding remarks

In conclusion, based on our clinical experience, it is not recommended to delay treatment in patients with acute severe UC who simultaneously present with COVID-19, even in the absence of respiratory complaints. Taking into account currently available evidence, we do not dissuade the use of steroids, calcineurin inhibitors, or TNF-α antagonists as induction therapy, and the use of TNF-α antagonists, vedolizumab or potentially ustekinumab as maintenance therapy in patients with acute severe UC and concurrent COVID-19 without respiratory complaints. Future research is warranted to investigate whether, and which, immunomodulatory and biological therapies should be used for symptomatic COVID-19 patients who clinically present with acute severe UC. Therefore, it is of utmost importance that future cases of patients with UC and COVID-19 are documented carefully in the worldwide SECURE-IBD registry.
